# Relationship between renal and liver function with diabetic retinopathy in patients with type 2 diabetes mellitus: a study based on cross-sectional data

**DOI:** 10.1038/s41598-022-13164-7

**Published:** 2022-06-07

**Authors:** Xi Yao, Xiaoting Pei, Shuoning Fan, Xueke Yang, Yingrui Yang, Zhijie Li

**Affiliations:** 1 Henan Eye Institute, Henan Eye Hospital, and Henan Key Laboratory of Ophthalmology and Visual Science, Henan Provincial People’s Hospital, Zhengzhou University People’s Hospital, No. 7, Weiwu Road, Zhengzhou, 450003 Henan China; 2 School of Public Health, Zhengzhou University, Zhengzhou, 450001 Henan China

**Keywords:** Retinal diseases, Risk factors

## Abstract

This study aims to explore the relationship between abnormal renal- and liver-function and diabetic retinopathy (DR) in patients with type 2 diabetes mellitus (T2DM). A total of 994 T2DM patients who received inpatient treatment in the Endocrinology Department of Henan Province People’s Hospital were included in the study. Logistic regression was performed to identify the relationship between abnormal renal and liver function with DR. Receiver operator characteristic analysis was performed to explore the efficacy of risk factors in predicting DR. Higher urine albumin [*OR*(95%*CI*) = 3.344(1.921–5.822), *P* < 0.001] and urine albumin/creatinine ratio [*OR* (95%*CI*) = 2.901(1.911–5.822), *P* < 0.001] were closely related to the occurrence of DR. People with low TP had a 1.624-times higher risk (95%*CI*: 1.008–2.617) of developing DR than those with normal total protein (*P* = 0.046). The more risk factors that are present, the greater the risk of DR. For every one-point incremental increase in the risk-factor score, the risk of DR increased by 31.0% (*P* < 0.001). The area under receiver operating curve of risk-factor score was 0.839 (0.812, 0.866), with a sensitivity of 81.9% and a specificity of 74.8%. The risk of developing DR increased with an increased risk-factor score. These findings are potentially valuable for DR screening and early diagnosis in patients with T2DM.

## Introduction

Diabetic retinopathy (DR) is a common complication of diabetes, and has been identified as the main cause of blindness in working adults. With the increasing prevalence of type 2 diabetes mellitus (T2DM), the impacts of DR also continue to rise, including damage to vision and the increased risk of cardiovascular disease^1^. It is estimated that the global prevalence of DR will increase to 5.4% by 2025^2^. Nearly 100 million adults in China suffer from T2DM, and DR has become one of the major public health problems in the world^3^. Studies have shown that delayed diagnosis of DR can lead to vision threatening complications, which means that it is necessary to provide sensitive and specific biomarkers for the early diagnosis of DR.

Both DR and diabetic nephropathy are diabetic microvascular complications, and the relationship between them has long been a popular area of study among researchers^4,5^. Genetic studies have shown that there is a common genetic susceptibility between DR and chronic kidney disease in diabetic patients^6^. The increase in the severity of DR is significantly associated with decreased renal function and increased risk of chronic kidney disease^7^, which may be caused by the same pathogenic mechanisms, including hyperglycemia-induced oxidative stress, advanced glycation end-product accumulation, increased reactive oxygen species, the abnormal activation of protein kinase C, and the abnormal activation of renin-angiotensin system. DR staging has been positively correlated with the staging of the glomerular filtration rate (GFR) and urinary albumin/creatinine ratio (UACR)^4^. Meanwhile, DR and kidney disease have the same degree of disease progression, and renal function-related indicators could be helpful in predicting DR^8^.

On the other hand, in people with diabetes, insulin resistance has been proven to cause dyslipidemia by increasing low-density lipoprotein cholesterol (LDL-C), total cholesterol (TC), free fatty acids and triglycerides, as well as by reducing high-density lipoprotein cholesterol (HDL-C) and inhibiting cholesterol reverse-transport genes^9,10^. Additionally, patients with diabetic dyslipidemia have a higher frequency of retinal abnormalities^11,12^. It has been reported that the level of lipoprotein (a), apolipoproteins, LDL-C and TC had strong correlation with the incidence and severity of DR^13^, which are likely linked by the influence of liver function. Increasing evidence shows that dyslipidemia is closely related to the risk of developing DR and cardiovascular diseases. However, in most related studies, no information is included about the renal status of subjects, which may be associated with liver dysfunction. Furthermore, there is limited information about the effects of the correlation between liver and renal dysfunction and retinopathy in T2DM patients.

The occurrence of disease is the result of a combination of multiple factors. As the types of risk factors accumulate, the risk of DR increases accordingly. Indeed, different types of risk factors contribute to the prevalence of DR to varying degrees. Considering the weight of risk factors for DR in analyses could more accurately reflect the essential relationship between risk factors and disease occurrence. Therefore, this study aims to explore the relationship between renal and liver function indicators and DR in T2DM patients, comprehensively analyze the correlation between risk-factor scores based on weight and the prevalence of DR, and determine the efficacy of risk-factor scores in predicting DR. These results could help endocrinologists predict the occurrence of DR through renal- and liver-function indicators, realize early diagnosis and treatment to delay disease progression, and eventually reduce disease burden.

## Methods

### Participants

A retrospective cross-sectional study was conducted with T2DM patients who received inpatient treatment in the endocrinology department of Henan Province People’s Hospital from May 2019 to May 2020. The inclusion criteria were as follows: (1) T2DM patients aged ≥ 18 years; and (2) completed ophthalmological clinical examination, such as fundus photography, image analysis, or retinal thickness analysis; The exclusion criteria included the following: (1) patients with hypertensive retinal disease, retinal vasculitis, retinal vein occlusion, or other retinal vascular diseases easily confused with DR; (2) patients with severe cardiopulmonary dysfunction, cerebral infarction, and other diseases; (3) patients without key variables (such as age, gender and diagnosis results); and (4) patients whose fundus photographs were unclear due to small pupils, cataracts, or vitreous opacity. Demographic characteristics, medical history, fundus examination results, and biochemical indicators of the included subjects were independently collected by two authors, and consistency checks were also simultaneously conducted. This study followed the Declaration of Helsinki and was approved by the Ethics Committee of Henan Provincial People’s Hospital (ethical approval code: HNEECKY-2022(22)). Written informed consent was obtained from all participants before enrollment.

### Related definitions and grouping standards

Patients were divided into two groups—diabetic retinopathy (DR) and no diabetic retinopathy (NDR) by two ophthalmologists with more than five years of work experience, according to the International Staging Standards for Diabetic Retinopathy in 2002^14^. (1) NDR: no obvious retinopathy and no abnormality; (2) mild non-proliferative diabetic retinopathy (NPDR): early stage of retinopathy with only microaneurysms; (3) moderate NPDR: some blood vessels that nourish the retinas are blocked; (4) severe NPDR: one or more of the following: (i) more than 20 intraretinal hemorrhages in each of the four quadrants of the retina, (ii) clear venous beading in two or more quadrants, and (iii) significant intraretinal microvascular abnormality in one or more quadrants; and (5) proliferative diabetic retinopathy (PDR): retinal signals triggering the growth of neovascularization in which the new blood vessels are abnormal and fragile. Fundus photographs of each participant were taken using a Zeiss non-mydriatic fundus camera (VISUCAM 224, Germany), with the macula as the center using 45° color-mode shooting. Five fields were captured in each eye: the macula center, temporal side, nasal side, and upper and lower quadrants of the retina^5^. Optical coherence tomography (OCT; Carl Zeiss Meditec, Germany) was used to observe the bergmeister papilla. Hyperuricemia (HUA): male-serum uric acid (SUA) concentration > 417 μmol/L, female-SUA concentration > 357 μmol/L^15^; High blood urea nitrogen (BUN): BUN > 8.3 mmol/L^16^. High urinary total protein (UTP): UTP ≥ 150 mg/24 hours^17^. Hypertension: systolic blood pressure ≥ 140 mmHg and/or diastolic blood pressure ≥ 90 mmHg^18^. Participant ages were divided into two groups: < 60 years and ≥ 60 years. The course of T2DM was divided into two groups: < 10 years and ≥ 10 years. High alanine aminotransferase (ALT) was defined as ALT ≥ 40U/L^19^. High aspartate aminotransferase (AST) was defined as AST > 40 U/L^20^. High cholinesterase (CHE) was defined as CHE > 10,500 U/L^21^. Low albumin (ALB) was defined as ALB < 35 g/L^22^. Low total protein (TP) was defined as TP < 60 g/L. High urine albumin (UALB) was defined as UALB > 200 mg/L^23^. High urinary albumin-creatinine ratio (UACR) was defined as UACR > 30 mg/g^24^.

### Statistical analysis

IBM SPSS Statistics (Version 23.0, SPSS Inc, Chicago, IL, USA) was used for statistical analysis in this study. Demographic and biochemical index of participants were described as means ± standard deviation (normal distribution data) or M (P_25_-P_75_) (skew distribution data) for quantitative variables, and as frequencies for qualitative variables. For qualitative data, the *χ*^2^ test was used to compare the differences in gender and hypertension distribution between two groups. For quantitative data, *t*-test or nonparametric test was used to compare the differences in biochemical index between two groups. Logistic regression analysis was used to identify the indicators that have an impact on DR. The weight of every risk factor was assigned according to the *OR* value. Logistic regression analysis was performed based on the number of risk factors and total risk-factor score. Receiver operator characteristic (ROC) analysis was conducted to determine the efficacy of risk-factor score in predicting DR in T2DM patients. All hypotheses were tested by two-sided, and the test level was *α* = 0.05.

### Ethics approval and consent to participate

The protocol for the study was approved by the Ethics Committee of Henan Provincial People’s Hospital [registration number (HNEECKY-2022(22))].

### Consent for publication

All participants and authors give consent for publication.

## Results

### General descriptions

A total of 994 diabetic patients were enrolled in this study, including 332 patients with DR and 662 patients without DR. There were 556 men and 438 women. The average age was 59.37 ± 13.29 years old. The average duration of diabetes was 9.51 ± 7.25 years. There were 488 patients with hypertension. There were statistically significant differences between the DR group and the NDR group in terms of gender, prevalence of hypertension, course of T2DM, renal (BUN, UALB, UCr, UACR) and liver (ALT, AST, ALB, and TP) function-related indicators (*P* < 0.05). The demographic information and biochemical indicators of the participants are shown in Table 1.

**Table 1:** General characteristics and biochemical indexes of the study population.

Variables	NDR (n = 662)	DR (n = 332)	Total N = 994	Statistics	*P*
Gender (Male/Female)	400/262	156/176	556/438	16.192^a^	< 0.001
Hypertension (Yes/No)	301/361	187/145	488/506	10.429^a^	0.001
Age (Years old)^d^	60 (49–68)	61 (52–69)	60 (50–68)	1.848^b^	0.065
HbA1c (%)^d^	9.40 (7.70–11.30)	9.40 (8.00–11.20)	9.40 (7.80–11.30)	1.034^b^	0.302
Duration (Year)^d^	7.00 (1.75–12.00)	10.50 (8.00–18.00)	10.00 (3.00–15.00)	9.387^b^	< 0.001
SUA (μmol/L)	363.25 ± 112.50	377.81 ± 127.92	368.05 ± 117.95	2.594^c^	0.068
BUN (mmol/L)^d^	5.40 (4.41–6.79)	6.14 (4.90–9.06)	5.60 (4.50–7.40)	4.371^b^	0.010
UTP (mg/24 h)^d^	94.67 (60.15–178.17)	154.76 (75.18–607.04)	109.79 (62.53–251.37)	1.372^b^	0.170
UALB (mg/L)^d^	7.01 (3.00–23.29)	34.41 (6.72–297.34)	9.34 (3.81–49.47)	6.841^b^	< 0.001
UCr(mmol/d)^d^	7.43 (4.64–12.12)	5.87 (3.89–8.49)	6.80 (4.21–11.06)	3.118^b^	0.002
UACR (g/mg)^d^	6.91 (3.57–26.14)	53.25 (9.82–521.78)	11.65 (4.30–85.87)	7.042^b^	< 0.001
ALT (U/L)^d^	19 (14–27)	16 (12–23)	17 (13–25)	4.484^b^	< 0.001
AST (U/L)^d^	20 (16–24)	17 (14–23)	19 (15–24)	4.158^b^	< 0.001
CHE (U/L)	8363.61 ± 2083.08	8219.52 ± 2225.56	8315.49 ± 2131.69	1.005^c^	0.315
ALB (g/L)^d^	38.15 (35.50–40.30)	36.50 (32.60–39.70)	37.60 (34.88–40.10)	5.060^b^	< 0.001
TP (g/L)^d^	65.85 (62.40–68.70)	65.30 (60.88–67.90)	65.60 (62.10–68.60)	2.394^b^	0.017

*NDR* non-diabetic retinopathy, *DR* diabetic retinopathy, *HbA1c* hemoglobin A1c, *SUA* serum uric acid, *BUN* blood urea nitrogen, *UTP* urinary total protein, *UALB* urine albumin, *UCr* urine creatinine, *UACR* urinary albumin-creatinine ratio, *ALT* alanine aminotransferase, *AST* aspartate aminotransferase, *CHE*: cholinesterase, *ALB* albumin, *TP* total protein.

a: chi-square value; b: non-parametric test statistic of the Z value; c: *t* value obtained from the t-test of two independent samples; d: data were described as median (inter-quartile range), that is M(P_25_, P_75_).

### Relationship between DR and renal function–related indicators

After converting the renal function–related indicators into binary variables, their relationships with DR were analyzed. The results showed that abnormal BUN, UTP, UALB, and UACR might have an impact on DR (*P* < 0.05). Stratified analysis was also conducted to identify confounding factors. The results showed that age might be a confounding factor for the effect of high UALB on DR. Gender might be a confounding factor for UALB and UTP affecting DR. Hypertension might be a confounding factor for high UALB and high UACR affecting DR. Furthermore, the course of T2DM might be a confounding factor for BUN and UALB affecting DR. Table 2 shows the *OR* values and 95% confidence intervals of abnormal renal function–related indicators and DR.

**Table 2:** The OR value and 95% confidence interval of abnormal renal function indexes and DR [*OR*(95%*CI*)].

	Hyperuricemia	High blood urea nitrogen	High urinary total protein	High urine albumin	High urine creatinine	High UACR
**Age (years old)**						
≤ 60	1.22 (0.84, 1.89)	2.76 (1.71, 4.43)	2.08 (1.42, 3.04)	12.74 (6.83, 23.75)	0.58 (0.31, 1.09)	5.32 (3.50, 8.08)
> 60	1.05 (0.72, 1.53)	1.87 (1.27, 2.74)	2.73 (1.85, 4.01)	4.01 (2.48, 6.42)	0.99 (0.98, 1.00)	4.80 (3.21, 7.18)
**Gender**						
Male	1.26 (0.87, 1.84)	2.32 (1.57, 3.42)	3.60 (2.45, 5.30)	8.00 (4.94, 12.95)	0.77 (0.39, 1.51)	5.41 (3.63, 8.06)
Female	0.94 (0.64, 1.38)	2.32 (1.46, 3.67)	1.64 (1.10, 2.44)	5.77 (3.15, 10.56)	0.24 (0.05, 1.08)	4.20 (2.79, 6.31)
**Hypertension**						
Yes	1.40 (0.97, 2.02)	2.45 (1.65, 3.65)	2.37 (1.63, 3.45)	4.41 (2.85, 6.83)	0.44 (0.19, 0.98)	4.20 (2.85, 6.19)
No	0.82 (0.54, 1.22)	1.69 (1.09, 2.62)	2.18 (1.46, 3.26)	18.12 (7.42, 44.30)	0.63 (0.25, 1.59)	5.45 (3.54, 8.37)
**Duration of T2DM**						
< 10 years	1.36 (0.88, 2.12)	2.29 (1.35, 4.88)	2.09 (1.33, 3.27)	9.59 (4.60, 19.99)	0.82 (0.40, 1.70)	3.65 (2.23, 5.98)
≥ 10 years	0.93 (0.65, 1.32)	1.43 (0.99, 2.06)	2.24 (1.56, 3.20)	4.13 (2.66, 6.41)	0.80 (0.22, 2.86)	4.05 (2.79, 5.88)
Total	1.14 (0.87, 1.49)	2.13 (1.59, 2.84)	2.38 (1.82, 3.12)	6.51 (4.49, 9.44)	0.53 (0.29, 0.97)	4.88 (3.67, 6.48)

### Relationship between DR and liver function–related indicators

After converting the liver function–related indicators into binary variables, their relationship with DR was analyzed. The results showed that low ALB and TP impact the prevalence of DR (*P* < 0.05). In stratified analysis, age and gender might have a mixed influence on the effects of ALB on DR. Age, gender, whether the patients had hypertension, and the course of T2DM might have a confounding influence on the effects of TP on DR. Table 3 shows the *OR* value and 95% confidence interval of abnormal liver function-related indicators and DR.

**Table 3:** OR value and 95% confidence interval of abnormal liver function index and DR [*OR*(95%*CI*)].

	High alanine aminotransferase	High aspartate aminotransferase	High cholinesterase	Low albumin	Low total protein
**Age (years old)**					
≤ 60	0.21 (0.10, 0.46)	0.32 (0.16, 0.64)	0.71 (0.44, 1.16)	3.88 (2.48, 6.08)	2.57 (1.57, 4.19)
> 60	1.63 (0.85, 3.13)	1.32 (0.83, 2.11)	0.68 (0.30, 1.56)	1.30 (0.88, 1.94)	1.21 (0.73, 1.99)
**Gender**					
Male	0.58 (0.30, 1.13)	0.87 (0.50, 1.50)	0.90 (0.52, 1.56)	2.89 (1.94, 4.28)	1.60 (1.03, 2.49)
Female	0.51 (0.27, 0.97)	0.65 (0.39, 1.09)	0.52 (0.28, 0.97)	1.60 (1.03, 2.50)	3.16 (1.67, 5.99)
**Hypertension**					
Yes	0.94 (0.52, 1.69)	1.06 (0.64, 1.75)	0.46 (0.27,0.79)	2.32 (1.55, 3.47)	2.65 (1.57, 4.48)
No	0.24 (0.10, 0.58)	0.54 (0.30, 0.98)	1.11 (0.59, 2.11)	1.86 (1.21, 2.85)	1.34 (0.82, 2.18)
**Duration of T2DM**					
< 10 years	0.45 (0.21, 0.96)	0.58 (0.30, 1.12)	0.81 (0.44, 1.48)	2.39 (1.50, 3,81)	1.64 (0.96, 2.79)
≥ 10 years	0.82 (0.44, 1.53)	0.99 (0.61, 1.63)	0.84 (0.46, 1.54)	2.01 (1.35, 2.98)	2.40 (1.43, 4.03)
Total	0.55 (0.35, 0.88)	0.78 (0.54, 1.13)	0.70 (0.46, 1.05)	2.11 (1.58, 2.83)	1.76 (1.25, 2.50)

### Logistic regression analysis of factors influencing DR

**Figure 1:**
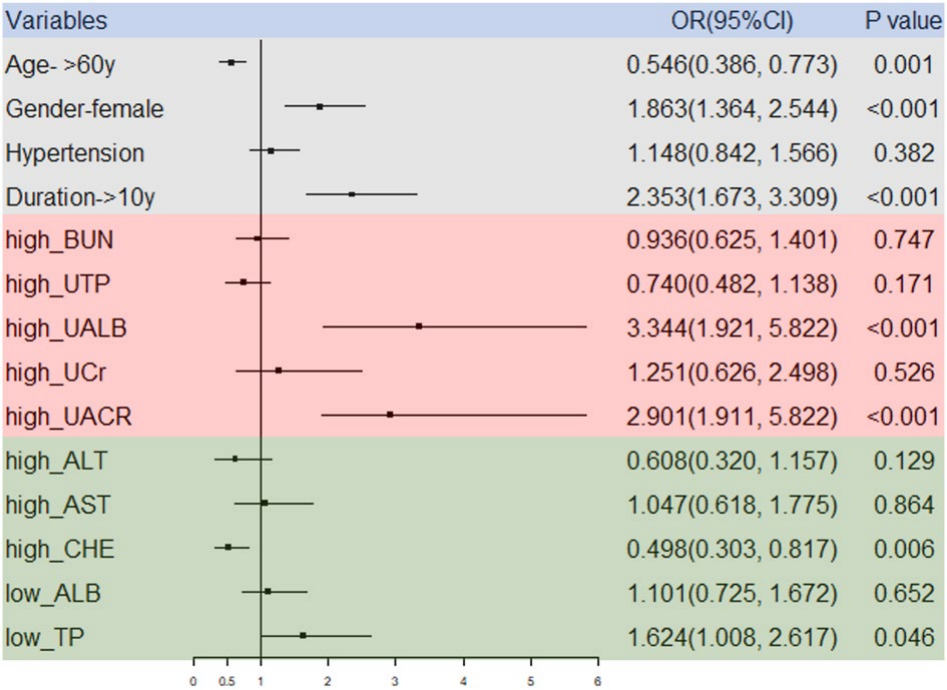
Logistic regression forest plot of DR and influencing factors. BUN: blood urea nitrogen; UTP: urinary total protein; UALB: urine albumin; UCr: urine creatinine; UACR: urinary albumin-creatinine ratio; ALT: alanine aminotransferase; AST: aspartate aminotransferase; CHE: cholinesterase; ALB: albumin; TP: total protein.

The renal- and liver-related indicators screened out by the univariate analysis were used as the independent variables, potential confounding factors (age, gender, hypertension status and T2DM duration) as the covariates, and DR status as the dependent variable; logistic regression analysis was then performed. The results showed that the DR risk of T2DM patients aged > 60 years was lower than that of T2DM patients aged ≤ 60 years [*OR* (95%*CI*) = 0.546(0.386, 0.773)]. Women had a 1.863-fold higher risk (95% *CI*: 1.364, 2.544) of developing DR than men. Patients with diabetes for more than 10 years, high UALB, and high UACR were at risk for DR (*P* < 0.05). The logistic regression analysis of factors influencing DR is shown in Fig. 1.

### Efficacy of risk factors in predicting diabetic retinopathy in T2DM patients

**Figure 2:**
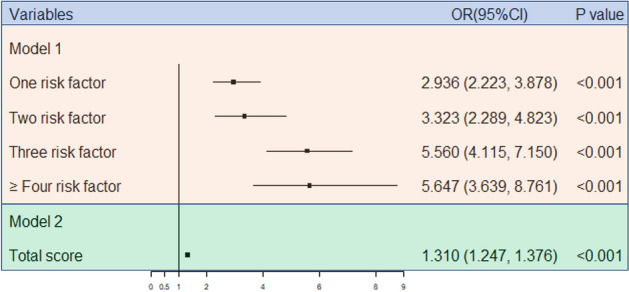
Logistic regression forest plot of combined risk factors and DR.

The aforementioned results showed that those aged ≤ 60 years old, women, and those with a duration of T2DM > 10 years, high UALB, high UACR, low TP, and low CHE were at risk for DR. Model 1: Taking the number of risk factors as the independent variables and the DR status as the dependent variable, logistic regression analysis was performed, and the *OR* values were calculated. Model 2: According to the *OR* values of the above results, the risk factors were assigned. The reference group was assigned to 0, and the rest of the groups were scored by rounding the nearest *OR* value. The scores were reversed if the *OR* value was less than 1. For example, the group with high UALB and high UACR were assigned to 3, and the group of women was assigned to 2. The logistic regression analysis was performed with the sum of the seven variables’ scores, and the *OR* values were calculated. The *OR* values of Model 1 and Model 2 are shown in Fig. 2. The results indicated that the more risk factors that are present, the higher the prevalence of DR. Compared to T2DM patients with risk factors < 4, the risk of DR increased by 4.647 times for diabetes patients with risk factors ≥ 4. In Model 2, for every one-point incremental increase in the score, the risk of DR increased by 31.0%.

**Figure 3:**
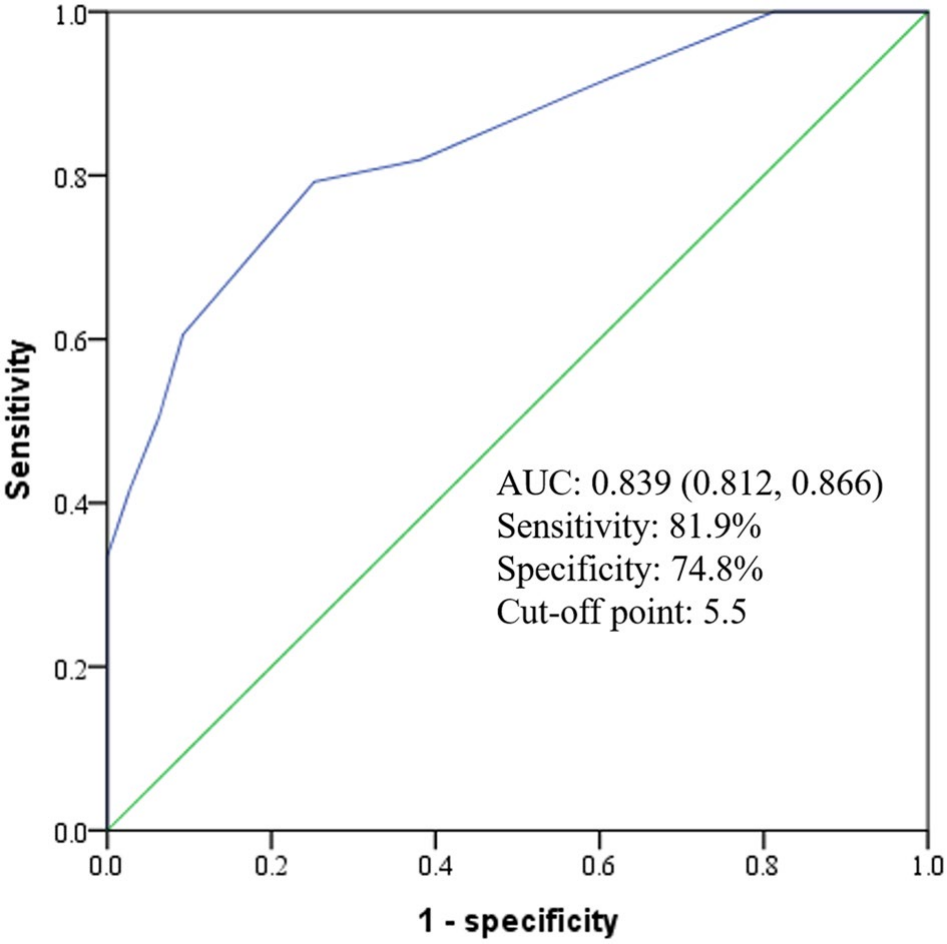
ROC curves for risk-factor score screening DR in T2DM patients.

ROC analysis was performed to explore the efficacy of risk-factor score in predicting diabetic retinopathy in T2DM patients, in which, DR was the state variable, and risk-factor score was the test variable. The ROC curve is presented in Fig. 3. The area under receiver operating curve (AUC) of the risk-factor score was 0.839 (0.812, 0.866). According to Youden’s index, risk-factor score > 5.5 were determined to be the best cut-off points for screening individuals with DR, with a sensitivity of 81.9% and a specificity of 74.8% (*P* < 0.001).

## Discussion

This study analyzed the relationship between renal and liver function–related indicators and DR in 994 T2DM patients and found the following: (1) abnormal renal and liver function-related indicators (high UALB, high UACR, low CHE, and low TP) might be risk factors for DR. The relationship between renal function–related indicators and DR was affected by age, gender, and the course of diabetes; (2) the more risk factors were present, the higher the weight-based score was, and the patients were also at higher risk of developing DR; (3) risk-factor score based on these indicators may be a valuable tool in screening for DR in T2DM patients.

The incidence of retinopathy was higher in patients with diabetic nephropathy than patients with diabetes alone^25^. The study by Pavel Kotlarsk et al. showed a positive relationship between renal damage and retinopathy in diabetic patients. A chronological order was also observed—namely, renal function damage occurred first, followed by retinal damage^26^. Thus, when abnormal renal–function indicators were detected, they not only indicated renal dysfunction but also the existence of DR. The examination of fundus was necessary for abnormal renal–function indicators in all diabetic patients. In addition, a study conducted in Sudan showed that hypertension, serum creatinine, and urea were risk factors for DR^27^. Another study in South Korea showed that diabetic retinal hyperplasia was related to microalbuminuria among patients with diabetes^28^. DR is regarded as a microvascular disease, and its main pathogenic links are closely related to pathological cascades^29^. Advanced DR is most associated with a high risk of cardiovascular disease^30,31^. Furthermore, the definition of the degree of diabetic retinopathy not only determines the risk of major fatal/non-fatal cardiovascular events (MACE) but also allows researchers and physicians to identify the subjects who could benefit from an intensified multifactorial intervention, both in terms of MACE and mortality, as was recently observed in the nephropathy of a T2DM study^32^. Therefore, the strict monitoring and control of blood glucose are key in preventing or delaying the occurrence of many cardiovascular diseases, especially for advanced DR patients.

On the basis of 994 T2DM patients in mainland China, this study showed that UACR was a risk factor for DR. The similar pathogenesis of DR and diabetic nephropathy might be partly explained by oxidative stress induced by hyperglycemia, the accumulation of advanced-glycation end products, increased production of reactive oxygen species, abnormal activation of protein kinase C, renal abnormal activation of the hormone-angiotensin system, etc.^4^. Moreover, the destruction of the glomerular filtration barrier could lead to high UACR^33,34^, followed by increased vascular permeability and aggravated damage of the blood–retinal barrier. The lipoprotein leaked into the extravascular space of the retina, and DR or diabetic macular edema (DME) eventually occurred^35^.

As a systemic metabolic disease, T2DM is often accompanied by abnormal lipid metabolism, including increased triglycerides, total cholesterol, and low-density lipoprotein, as well as decreased high-density lipoprotein^36^. Diabetic patients with DR were closely related to dyslipidemia^37^. Elevated serum apolipoprotein III, apolipoprotein E and apolipoprotein A-I were risk factors for DR^38^. High total cholesterol and triglycerides also increased the risk of DR. When all types of lipids were abnormal, the possibility of DR increased significantly^39^. The lipid metabolism process was closely related to liver function. In addition, it has recently been observed via liver biopsy that steatohepatitis represents the sole feature of liver damage in T2DM. This observation confirmed the hypothesis that T2DM and insulin-resistance status increase the risk of advanced fibrosis, with consequent worsening of both hepatic and cardiovascular outcomes^40^. In the future, the mediating effect of steatohepatitis in the occurrence of DR should be studied. Our results showed that the risk of DR in T2DM patients with low CHE and TP were higher than that of patients with normal CHE and TP, respectively. Therefore, the changes in blood lipids, CHE and TP of T2DM patients should be given significant attention, and fundus examination should be done promptly to ensure timely treatment measures are taken and to avoid the occurrence or development of DR.

This study showed that women had a 1.863-fold higher risk of developing DR than men, which was consistent with the results of the study by Zhuang^4^. Even after being stratified by age, hypertension status, and the course of diabetes, women were still at higher risk for DR^41^. Furthermore, 17β-estradiol (E2) was proven to protect retinal ganglion cell (RGC)-5 from high glucose-induced damage through the mitochondrial pathway^42^. Existing studies have suggested that estrogen has a protective effect on the occurrence of T2DM and DR. This study found that most of the female DR patients were between 50 and 70 years old [M (P25-P75): 55.5 (64.0–70.5) years old]. At this time, they were in the postmenopausal period, and estrogen was decreased, which might be a reason for the high incidence of DR in women.

Patients with diabetes > 10 years had a 2.353-times higher risk (95% *CI*: 1.673, 3.309) of developing DR than those with diabetes ≤ 10 years. Thus, the longer diabetes lasts, the more the effects of risk factors accumulate and the greater the risk of various DM-related chronic complications becomes. Interestingly, the risk of DR in T2DM patients ≤ 60 years old was actually higher than that of patients > 60 years old. Research by Li et al.^41^ also showed that the risk for DR increased with age for people < 60 years old, while the prevalence of DR decreased with age for people > 60 years old, which may be due to the mortality rates of DM in populations > 60 years old being higher than those in younger patients; thus, the prevalence of DR was relatively low in the research subjects. In addition, people ≤ 60 years old paid more attention to their health and tended to have regular fundus examinations, and thus the higher detection rate of DR in the younger populations could be explained.

The results of the logistic regression showed that the more risk factors were present in T2DM patients, the higher the prevalence of DR was. For each one-point incremental increase in the risk-factor score based on weight, the risk of DR increased by 31.0%. After five years’ follow up, Jeng’s study found that with the increase in the course of disease, changes in UALB, and UACR, as well as other indicators, the risk of DR in patients with diabetes nephropathy was 2.26-times higher than that of patients with general diabetes^25^. Jin et al*.*^43^ confirmed that the simultaneous occurrence of hyperglycemia and hypertension increases the risk of DR in diabetes patients. It was clear that the occurrence and progression of DR was the result of a combination of multiple risk factors. We found that the weight-corrected risk-factor score could be a valuable tool for predicting DR in T2DM patients, with an AUC of 0.839 (0.812, 0.866), sensitivity of 81.9%, and specificity of 74.8%. Therefore, a holistic medical approach, regular screening for multiple risk factors, and comprehensive interventions are needed in treating patients with DR.

With the development of the Internet and 5G technology, artificial intelligence and telemedicine have become the focus of much research, especially in areas with poor medical technology and developing countries^44,45^. Data from the 21st National Ophthalmology Conference of the Chinese Medical Association held in 2016 indicated that 20% of county hospitals in China did not have an ophthalmology department. Even in institutions with such a department, there were few specialists in fundus diseases. The ratio of ophthalmologists to patients was 1:3000 in these areas^46^. Adding the results of this research into the deep-learning algorithm of artificial intelligence would further the present study. With telemedicine based on artificial intelligence, it was possible to carry out a large-scale screening for high-risk groups and outpatients, particularly during the current pandemic^47^.

However, this study also had several limitations. First, the research subjects came only from a single hospital in Henan Province. Due to the discrepancies in the level of diagnosis and treatment, living habits, and diagnostic criteria in different regions, the results of this study might not be generalizable to other regions or populations. Second, the study was cross-sectional, and it was impossible to determine whether there was a causal relationship between abnormal renal and liver function-related indicators and DR. Third, this study lacked differentiation between the various degrees of DR; furthermore, the level of renal and liver function-related indicators and risk of complications are associated with the grading of DR, which may affect the accuracy of the results. Finally, the participants included in this study were all T2DM patients receiving inpatient treatment in the Endocrinology Department; thus, they may be different from T2DM patients within the general community. Thus, the correlation between abnormal renal- and liver-function indicators and DR might have been overestimated. Therefore, large prospective studies based on multiple regions are needed to verify this relationship.

## Conclusions

There was a correlation between abnormal renal and liver function and DR, which might be affected by age, gender, hypertension status, and diabetes course. The risk of developing DR increased with the number of risk factors or higher risk-factor score. The weight-corrected risk-factor score could be a valuable tool for predicting DR in T2DM patients. These findings are helpful for the early diagnosis of DR and improvement in quality of life for patients with DR.

## Data Availability

All relevant data are included in the papers. Contact to corresponding author for additional information regarding data access.

## Abbreviations

DR: Diabetic retinopathy
T2DM: Type 2 diabetes mellitus
GFR: Glomerular filtration rate
UACR: Urinary albumin/creatinine ratio
NDR: No diabetic retinopathy
HUA: Hyperuricemia
SUA: Serum uric acid
BUN: Blood urea nitrogen
UTP: Urinary total protein
ALT: Alanine aminotransferase
AST: Aspartate aminotransferase
CHE: Cholinesterase
ALB: Albumin
TP: Total protein
UALB: Urine albumin
UACR: Urinary albumin-creatinine ratio
ROC: Receiver operator characteristic
UCr: Urine creatinine
AUC: Area under receiver operating curve
RGC: Retinal ganglion cell
E2: Estradiol
LDL-C: Low-density lipoprotein cholesterol
TC: Total cholesterol
HDL-C: High-density lipoprotein cholesterol
MACE: Major fatal/non-fatal cardiovascular events
DME: Diabetic macular edema
DM: Diabetes mellitus
